# What Does “Good” Community and Public Engagement Look Like? Developing Relationships With Community Members in Global Health Research

**DOI:** 10.3389/fpubh.2021.776940

**Published:** 2022-01-27

**Authors:** Gary Hickey, Katie Porter, Doreen Tembo, Una Rennard, Martha Tholanah, Peter Beresford, David Chandler, Moses Chimbari, Tina Coldham, Lisa Dikomitis, Biggy Dziro, Peter O. Ekiikina, Maria I. Khattak, Cristian R. Montenegro, Noni Mumba, Rosemary Musesengwa, Erica Nelson, Clement Nhunzvi, Caroline M. Ramirez, Sophie Staniszewska

**Affiliations:** ^1^Wessex Institute, Faculty of Medicine, University of Southampton, Southampton, United Kingdom; ^2^School of Health Sciences, University of Brighton, Brighton, United Kingdom; ^3^Public Contributor, Oxfordshire, United Kingdom; ^4^AIDS Clinical Trial Group Clinical Research Site Community Advisory Board, Clinical Trials Research Centre, University of Zimbabwe, Harare, Zimbabwe; ^5^Making Waves Network, Harare, Zimbabwe; ^6^School of Health Sciences, University of East Anglia, Norwich, United Kingdom; ^7^The Psoriasis and Psoriatic Arthritis Alliance, St Albans, United Kingdom; ^8^School of Nursing and Public Health, College of Health Sciences, University of KwaZulu-Natal, Durban, South Africa; ^9^Department of Public Health, Great Zimbabwe University, Masvingo, Zimbabwe; ^10^National Institute for Health Research Centre for Engagement and Dissemination, London, United Kingdom; ^11^School for Social Care Research, National Institute for Health Research, London, United Kingdom; ^12^Kent and Medway Medical School, University of Kent and Canterbury Christ Church University, Canterbury, United Kingdom; ^13^African Mental Health Research Initiative (AMARI), Harare, Zimbabwe; ^14^Foundation for Open Development, Tororo, Uganda; ^15^Institute of Public Health & Social Sciences, Khyber Medical University, Peshawar, Pakistan; ^16^Wellcome Centre for Cultures and Environments of Health, University of Exeter, Exeter, United Kingdom; ^17^Escuela de Enfermería, Pontificia Universidad Católica de Chile, Santiago, Chile; ^18^KEMRI Wellcome Trust Research Programme, Kilifi, Kenya; ^19^Medical Sciences Division, Department of Psychiatry, University of Oxford, Oxford, United Kingdom; ^20^Health and Nutrition Cluster, The Institute of Development Studies, University of Sussex, Brighton, United Kingdom; ^21^College of Health Sciences, University of Zimbabwe, Harare, Zimbabwe; ^22^School of Medicine and Public Health, Ateneo de Manila University, Pasig, Philippines; ^23^Harvard Medical School, Boston, MA, United States; ^24^Warwick Research in Nursing, Warwick Medical School, University of Warwick, Coventry, United Kingdom

**Keywords:** patient and public involvement, research relationships, power dynamics, research stakeholders, respecting community

## Abstract

Community and public engagement (CPE) is increasingly becoming a key component in global health research. The National Institute for Health Research (NIHR) is one of the leading funders in the UK of global health research and requires a robust CPE element in the research it funds, along with CPE monitoring and evaluation. But what does “good” CPE look like? And what factors facilitate or inhibit good CPE? Addressing these questions would help ensure clarity of expectations of award holders, and inform effective monitoring frameworks and the development of guidance. The work reported upon here builds on existing guidance and is a first step in trying to identify the key components of what “good” CPE looks like, which can be used for all approaches to global health research and in a range of different settings and contexts. This article draws on data collected as part of an evaluation of CPE by 53 NIHR-funded award holders to provide insights on CPE practice in global health research. This data was then debated, developed and refined by a group of researchers, CPE specialists and public contributors to explore what “good” CPE looks like, and the barriers and facilitators to good CPE. A key finding was the importance, for some research, of investing in and developing long term relationships with communities, perhaps beyond the life cycle of a project; this was regarded as crucial to the development of trust, addressing power differentials and ensuring the legacy of the research was of benefit to the community.

## Introduction

Community and public engagement (CPE) in the development, undertaking and delivery of global health research, interventions and policy is increasingly regarded as essential by funding bodies (1–3). We use CPE for the purposes of this paper, but the term officially used and referenced by NIHR is community engagement and involvement (CEI). The National Institute of Research (NIHR) is committed to CPE and to involving the most marginalized communities in the global health research it funds, arguing that it is vital to improving the reach, quality and impact of the research. The recognition of the importance of CPE has led to the development of various guidelines and standards for CPE generally (3, 4), techniques and approaches for CPE, such as approaches guided by participatory action research techniques (5), and CPE criteria being included in ethical guidelines that apply to global health research specifically (6–13).

The UK equivalent of CPE is patient and public involvement (PPI). The UK Public Involvement Standards Development Partnership guidance on “what good looks like” in PPI has been encapsulated in the six standards for public involvement (14). These standards are not a prescriptive “how to” manual; they can find expression in a variety of ways and can be used to guide and evaluate PPI in research. Furthermore, they are flexible enough to be applied to all research topic areas and in conjunction with any research methods.

As the NIHR further develops a portfolio of work in global health, what can its past experience championing PPI contribute to current debates on what constitutes “good” CPE? And can we develop something that involves a partnership of actors from both high income countries (HICs) and low and middle income countries (LMICs)? The CPE guidelines that are currently available are useful, but many are either non-health research focused (3), focused on a specific region/condition or research approach (4, 15, 16), or focused on the ethics of engagement (17). Clear guidance on CPE, which builds on existing guidelines and frameworks, would be useful in ensuring clarity of expectations of award holders, and the design of monitoring and evaluation frameworks. Of course, it must take account of the reality that CPE is not free-standing and is likely to be affected by the nature of politics and policy drivers in any particular setting (18).

The NIHR, in collaboration with the UK's Institute of Development Studies, has recently produced a series of learning resources to support applicants and researchers in planning and delivering meaningful CPE (1). With this paper, we hope to add to and build on this resource. This paper is our first step in trying to identify the key components of what makes for “good” CPE, which can be applied across all approaches to global health research as well as different countries and contexts.

## Methods

Thematic data analysis (19) of 139 progress reports submitted between 2017 and 2019 by all 53 NIHR Global Health Research Units and Groups was undertaken by two members of the study team (Table 1). The UK-led Units and Groups deliver world-class applied global health research and work in partnership with researchers in LMICs, who are eligible to receive UK funding, to address under-funded or under-researched topics specific to those countries (20). At the time of writing, the Units and Groups involved in this analysis have either completed or are nearing completion of their funded research. Inductive coding was used to identify common themes (19) highlighting potential enablers for and barriers to good CPE. Qualitative data analysis was supported by NVivo software.

**Table 1:** Further information on the sample used in the content analysis and the participants involved in the workshop.

**Method**	**Sample**
Content analysis	No Units or Groups (that were funded at the time of analysis) were excluded. No available progress reports were excluded. The Units and Groups that were included in the content analysis were collectively undertaking research in 61 LMICs, as follows: - 12 LMICs in Latin America and the Caribbean - 2 LMICs in Northern Africa - 23 LMICs in Sub-Saharan Africa - 4 LMICs in Middle East - 1 LMIC in East Asia - 5 LMICs in South Asia - 10 LMICs in Southeast Asia - 4 LMICs in Europe
Workshop	Purposive sampling was used to identify a group of CPE/PPI specialists and public contributors located in diverse country contexts that could bring a range of experience to the workshop. Out of the 18 people who were invited to participate, 11 were able to attend. The 11 workshop participants are authors on the paper along with the 7 people who could not attend but were involved in other aspects of the research. The global regions represented in the workshop, and the number of participants from each of these contexts were: - UK (7) - Sub-Saharan Africa (2) - Southeast Asia (1) - South Asia (1)
	Workshop attendees included seven people who would be considered CPE and/or PPI specialists and four public contributors with lived experience.

The findings from the content analysis informed the discussion at a workshop where participants explored what good CPE looks like and identified factors that facilitate and inhibit CPE. The workshop was attended by 11 participants and facilitated by two representatives of NIHR (Table 1). Participants broke up into two groups, and each group addressed questions relating to enablers and barriers of CPE. Discussions were transcribed after the workshop via an online transcription service, and quality checked by two members of the research team by listening to the recordings. Common themes were identified from the workshop transcript through use of inductive coding (19) by two members of the research team.

## Emergent Findings

The potential enablers of good CPE that emerged from the content analysis and subsequent workshop discussion are presented in Table 2. These were merged to form broad potential enablers which are outlined in the next section and interpreted in light of the literature in this area. In practice, these enablers are not exclusive, but rather they overlap and intertwine to make up what “good” looks like in CPE.

**Table 2:** Potential enablers of good community and public engagement (CPE) as identified through the content analysis, workshop discussions and the merged findings of these two processes.

**Enablers from the content analysis**	**Enablers from the workshop discussion**	**Merged potential enablers of good CPE**
Knowledge of community dynamics and structure	Respond and adjust to cultural norms, and increase cultural competence of researchers	Adaptation to local cultural norms and customs
Awareness and knowledge of the research amongst the community members involved		
Create opportunities for open communication and feedback	Avoid transactional relationships and encourage open and honest communication	Treat community members with respect
Respond and adjust to the barriers to involving marginalised communities in research	Actively reach out to the community	
	Respect the diversity of local knowledge and reflect on hierarchies of knowledge at the local level	
Awareness of local gatekeepers and when they might restrict access to community members	Understand how to work with gatekeepers and why they might restrict access to community members	Acquire permission from and work with local gatekeepers
Awareness of power inequities between HIC researchers and the LMIC community members (as well as between community members)	Identify and address power inequities within and between local communities	
Community involvement from the outset to ensure relevance of research to the local context	Undertake research that is relevant to the community and involve them in developing research priorities	Seek community involvement in, and ownership of, the research
Undertake locally led activities in the health intervention with the community		
Involve multiple local stakeholders to ensure the intervention is beneficial to all		
Encourage development of community members and their engagement with issues (aka a “virtuous circle”)		
Utilization of strong existing relationships when available to quickly get CPE activities started	Avoid overburdening communities (i.e., different research teams involving the same community members over an extended period of time)	Avoid overburdening communities
Address competing research priorities e.g., policy makers vs. local communities vs. HIC researchers Understand how CPE activities are restricted by finite resourcing and funding	Investment in long term relationships (or the legacy of the research) to enable partnerships which address research and community needs around social justice and long term health outcomes	Investment in long term relationships and research goals

## Discussion

### Adaptation to Local Cultural Norms and Customs

The importance of being aware of and sensitive to cultural and social differences is a key principle of ethical CPE (10, 11). This is underpinned by the notion of respecting cultural differences—which is addressed in the next section. The example below shows how awareness of, and adjusting CPE activities to fit, local culture and community dynamics can lead to the inclusion of people who otherwise would not be part of research.

“*In Pakistan and Bangladesh, engagement of women in the research can be challenging, but is overcome by having dedicated facilities (or sessions) for women, where they are seen by female only staff. In contrast, in Sri Lanka, engagement of men is harder, as they place their main focus on their employment. We overcome this by adopting approaches that more actively engage with men, approaching employers to release their workers for health assessments / interventions and by making sessions outside the working day (evenings and weekends).” (Unit #6 - from content analysis)*

Respecting and adapting to local cultural norms and customs also finds expression in researchers traveling to reach the community. Expecting community members to travel to academic institutions can exacerbate the perceived power imbalances between the researchers and the community (6, 21), and so engaging people within their community context can make them feel more comfortable in conversations.

When explored further in the workshop, researchers dressing in a certain way was given as a further example of adaptation.

“*…we need to accept certain cultural norms, for example, I'll share from my experience, I don't (usually) cover my hair, I don't wear a headscarf…. If I go into a suburb or rural area, I have to change the way I dress up” (Workshop participant #5)*

The excerpt below demonstrates that adapting also applies to incentives to participate in research. Researchers should be conscious that what is considered morally and ethically acceptable may differ across cultures and countries (22).

“*But I was so surprised when for the first time I went to the Philippines, … the degrees on the wall, … you know, like mayors and, and government officials. But they were not degrees, they were kind of tokens and certificates of participation in a project. So then I started realising ‘Oh, I didn't bring anything'… The next time we went, we made sure that we did.” (Workshop participant #3)*

### Treat Community Members With Respect

The development of respect toward communities is another issue that is articulated in various ethics criteria (7, 11, 23). This was an issue that was implied in the progress reports and addressed in detail in the workshop.

Respect found expression in terms of valuing local skills and knowledge. Gautier et al. (24) stress the importance of moving away from paternalistic, top-down CPE methods and encouraging listening and response methods between the researchers and the community. This sense of a two-way interaction, and valuing and respecting different types of knowledge, was discussed in our workshop.

“*…It's not bi-directional. It's just like one direction, assuming that someone knows more, and someone knows less. So someone has skills, all of these research competencies, you know, all of these degrees, and then someone has less, but how do we elevate the knowledge, the competencies, the skills of these people, and recognise them as valuable as what other people know and have? And I guess that's where the respect comes in as well and not having that kind of paternalistic approach…” (Workshop participant #9)*

Respect also includes appreciating and listening to local knowledge about the relationships and power dynamics within the community and relations with other communities in the area. Talking and listening to community members or local researchers can help non-local researchers to avoid tense situations.

“*…if somebody from the community goes into the community or understands the politics, the social economic dynamics, then that person would be able to understand not to bring these two tribes together, because that would be an all out war in that community engagement programme.” (Workshop participant #5)*

### Acquire Permission From, and Work With, Local Gatekeepers

The importance of engaging with local, regional and national health authorities (8) and gaining the necessary legitimacy via the permission and approval of local actors (22, 23, 25) was evident in the literature.

The content analysis and workshop discussions demonstrated that when engaging a community, researchers may have to work with local community leaders (i.e., gatekeepers) to gain access to a community or to get approval to carry out research in their area and give the research legitimacy.

“*trying to engage the community without engaging the local health ministry was a non-starter completely… there was a lot of inducements that needed to be applied to the local policymakers, and involved numerous meetings, numerous visits to the health ministry, basically tried to convince them, this is a good idea.” (Workshop participant #1)*

The workshop discussion also showed that community leaders were sometimes instrumental in creating barriers to working with the most marginalized communities. There are multiple reasons that gatekeepers might block entry to researchers, which can be predicated on past experiences with international or other forms of health research where they live.

“*…gatekeepers of or leaders of communities may restrict access to the most marginalised members of the community. And I think that's absolutely true (…) But a lot of it is not being paternalistic, but they are sometimes advocating for those members and keeping them safe.” (Workshop participant #2)*

There can also be a less benign side to some of those actors—political actors—whom researchers depend on for permission to do their research in the community. So, gatekeepers can be barriers as well as people who can facilitate access.

“*I had to cancel one of my events, because I was working with one member of parliament coming from an opposition political party. And when the government noted that, they withdrew the police services to cover my event.” (Workshop participant #7*).

### Seek Community Involvement in, and Ownership of, the Research

The importance of the community having ownership of the research and its outcomes emerged from analysis of the progress reports.

“*To ensure long-term, sustainable change, the local community has to voice the local concerns and participate in defining the healthcare challenges. In turn, we aim for communities to develop a sense of responsibility and ownership of the solutions.” (Unit #10 - from content analysis)*

The above excerpt hints at the notion of the “legacy” of the research. We define “legacy” as a concept that synthesizes the idea of sustainability and long-term impact; working toward the creation of long-term improvements that extends beyond the research lifecycle and creates a sense of ownership over the research within communities.

Explored further in the workshop, it was asserted that aligning the research with the communities priorities will keep it relevant to the local context and, ultimately, more likely the resulting intervention will be sustainable. Our findings support literature that show how involving people in the research can help ensure the relevance of the research to local communities (11, 12) as well as the development and maintenance of trust in the research from the local community (7, 11).

Workshop participants also explored the importance of involving local people in the research, which bestowed a degree of legitimacy on the research. The suggestion is that this can help promote consent to participate in the research (7, 22, 26).

“*…. bringing people in from outside that don't match maybe local profiles or local needs, will only alienate people. This is why peer to peer involvement is always so good. Because if one of your group can talk to you about something that they feel is important, then you're more likely to listen to them than to somebody else…” (Workshop participant #1)*

### Avoid Overburdening Communities

Avoiding exploiting people (9, 11, 23), ensuring the protection of participants (25) and making sure that communities are not overburdened (22) all feature in the literature. Overburdening communities, in terms of going back to the same community rather than reaching out to other communities, was an issue that emerged from the workshop.

“*…one thing that we should watch out for that I've seen happening, the University Department gets into a community (…) so anyone who is now going to do research keeps going to that same particular community. Even though there are other areas within let's say, in Harare, they will go to one particular suburb and just engage in work with that community. So then some are saying we are tired of these people.” (Workshop participant #13)*

### Investment in Long Term Relationships and Research Goals

Ensuring that research benefits the community is an often cited goal of CPE (7, 10, 25). Echoing the work of Pratt (27), workshop participants queried what or whose goals were the priority; the goals of the relatively short term research or the longer term goals of the community.

“*Whose rights are we prioritising? Is there kind of, you know, premium for what the community needs? And what do they say they need? Is that above, you know, whatever research or academic or even policy and goals there are.” (Workshop participant #9)*

Researchers should be mindful of the particular colonial and imperial histories that have shaped past public health interventions and practices in the geographic contexts in which they are working (6, 7, 27–29).

“*I think it's important to consider colonial history… and having that kind of paternalistic relationship, we know long term might not be the healthiest for us, for example.” (Workshop participant #9)*

An obvious example of how power inequities can find expression is in the language used between the community and the researcher, and also between researchers in HICs and LMICs.

“*I have to speak better English to talk to you – we take on the burden of adjusting ourselves to your system, your protocols.” (Workshop participant #9)*

Long term relationships, that went beyond the scope of a single project or funding cycle, were regarded as a key component of the development of trust, addressing power differentials and ensuring the community has real influence.

“*…I think it's a bit of a challenge when you don't have those existing community relationships and having to develop them fast can feel really uncomfortable, because you know, that you're hurrying people along, and you're not doing it in the way you would want to because, you know, Global Health bid come out, and you've got six weeks to deliver it.” (Workshop participant #11)*

This echoes Nelson's (6) assertion that establishing the foundations necessary for long term relationships does not always sit easily with short term fundings cycles. NIHR has recently set up funding arrangements to support the development of research applications and partnerships; it encourages early involvement of community members and the development of relationships between researchers and the community (30).

The sustainability of relationships between the community and researchers was regarded as a key component in ensuring the legacy of the research and this finding echoes the work of others (4, 16).

## Conclusions

Despite the volume of literature on CPE, there is no explicit CPE guidance that researchers can turn to for answers about what “good” CPE looks like and why it should be done. This paper is the first step on the path toward identifying what “good” CPE might look like. The enablers we have highlighted in our discussion have been drawn from the analysis of progress reports and a workshop which covered examples of CPE from multiple countries and a broad range of research areas.

Global health research is still largely led by academics based in HICs where the social, cultural and economic context is likely to be very different from LMICs (23, 26). Therefore, any guidance on CPE should give due consideration and respect to local cultures, as well as encouraging the development of trusting relationships with a variety of stakeholders to adapt the research to the local context. Embracing close relationships with community members throughout the research process can create channels for open communication and ensures that the research is responsive to the needs of the community (31).

Establishing long term relationships between researchers and community members was a key enabler of good CPE that emerged from our work. Clearly, researchers need to be mindful of overburdening sections of the community and sometimes long term relationships may not be feasible or desirable. The suggestion was, however, that relationships sometimes needed to be built beyond the time frame of a single project or research cycle, and only then could trust be sufficiently developed and power differentials addressed. This approach will better ensure that research is focussed on the goals and needs of the community rather than just that of the researchers or funders.

This work was led by a UK-based research funder. Any future work in developing the core components of “good” CPE must ensure that it continues to be done in partnership with, and draws on the knowledge and experiences of, people from LMICs. We intend to explore our emerging enablers further with key stakeholders with a view to further develop our ideas, and possibly guidance, on what constitutes “good” CPE.

## Data Availability Statement

The raw data supporting the conclusions of this article will be made available by the authors, upon request, without undue reservation.

## Author Contributions

MT, UR, DT, DC, TC, LD, MK, RM, CN, CR, and SS took part in the workshop discussion. GH and KP led the workshop discussion. GH, KP, UR, MT, and DT contributed to the analysis of the results and to the writing of the manuscript drafts. KP attests that all listed authors meet authorship criteria and that no others meeting the criteria have been omitted. All authors contributed equally to the design and implementation of the research, contributed to manuscript revisions, read, and approved the submitted version.

## Funding

SS was part funded by the NIHR Applied Research Collaboration (ARC) West Midlands, the NIHR Health Protection Research Unit (HPRU) Gastrointestinal Infections, and the NIHR HPRU Genomics and Enabling data. PB's time was supported by the National Institute for Health Research Applied Research Collaboration East of England (NIHR ARC East of England).

## Conflict of Interest

The authors declare that the research was conducted in the absence of any commercial or financial relationships that could be construed as a potential conflict of interest.

## Publisher's Note

All claims expressed in this article are solely those of the authors and do not necessarily represent those of their affiliated organizations, or those of the publisher, the editors and the reviewers. Any product that may be evaluated in this article, or claim that may be made by its manufacturer, is not guaranteed or endorsed by the publisher.
